# The mobility paradigm in higher education: a phenomenological study on the shift in learning space

**DOI:** 10.1186/s40561-021-00162-x

**Published:** 2021-08-30

**Authors:** Dishari Chattaraj, Arya Parakkate Vijayaraghavan

**Affiliations:** Department of English and Cultural Studies, Christ University, Bangalore, India

**Keywords:** COVID-19, Embodied skill acquisition, Higher education, Interpretive phenomenological study, Learning space, Mobility paradigm, Spatial shift

## Abstract

The study, through the framework of mobility and space, explores the phenomenon of multiple shifts in learning spaces induced by COVID-19. The Interpretative Phenomenological Approach (IPA) is adopted to document the experiences and perceptions of learners caught within these spatial shifts—physical, online, and hybrid. Online interviews were conducted with six first-year undergraduate and three first-year postgraduate students enrolled at the department of English and Cultural Studies in a Southern Indian University. Some of the dominant patterns emerging from the accounts of the participants are (1) the changing perception of conducive learning space, (2) the changing perceptions and roles of various classroom actors, and (3) the evolving nature of the learners and the learning process. The study utilizes the framework of mobility to locate the stage of embodied skill acquisition of the participants within the online learning space and illuminates the possibilities offered by this paradigm within the context of higher education. Some of the insights gained through the study include a changing perception of the conventional built classroom space, a notable preference towards a complete online or offline mode as opposed to the hybrid mode, and a transition towards self-directed learning. The study argues that these implications are highly pertinent and can significantly shape the way pedagogues and researchers engage with the various modes of learning—physical, online, and hybrid—and the future of higher education that is shaped by technology-enabled learning.

## Introduction

The shift to Emergency Remote Learning (ERL) as a response-measure to the pandemic COVID-19 changed the way learning was experienced in the pre-pandemic context. This unplanned and abrupt shift from architecturally planned learning contexts like schools, colleges, and universities to unplanned study-rooms and drawing-rooms of learners led pedagogues and researchers to evaluate the impact of this shift on the future of learning (Neuwirth et al., 2020; Rashid & Yadav, 2020). In the span of one year, educationists and scholars closely engaged with this ‘disruption’ and sudden transition to technology-enabled-learning by deliberating on 1. the future and new wave of digitized education (Bonfield et al., 2020), 2. the emerging pedagogical approaches, and assessment strategies (Huang et al., 2020; Murphy, 2020), 3. the accessibility and digital divide resulting from the shift (Neuwirth et al., 2020), and 4. the built environment, and novel complex learning spaces (Deshmukh, 2021; Goodyear, 2020).

As the pandemic and the subsequent lockdown and unlocking across the globe have been extending for over a period of one year, online and hybrid learning, as predicted, are becoming the new normal (Kaufman et al., 2021). The normalization of the new modes of learning has led to discussions on the need to renovate and reimagine existing infrastructure (Deshmukh, 2021), and debates over whether this shift in learning is a boon or a bane for higher education (Ma et al., 2021). While the spatial shifts are discussed, the transitions from the classroom spaces to home spaces, and partial transition back to the classroom spaces, have hardly been the focal point of inquiry. The present study, therefore, foregrounds these spatial shifts by tracing the recent movements towards the mobility paradigm in the field of education. Relatively a new paradigm of inquiry, the engagement with mobility is complex owing to the historical disengagement with space beyond the conventional built spaces in the field of education (Cox, 2018; Ellis & Goodyear, 2016; Gulson & Symes, 2017), especially considering the interconnectedness between spatial studies and the mobility paradigm (Sheller, 2017). Through the framework of mobility and space, the present study attempts to understand the phenomenon of multiple spatial shifts and its impacts on the perception and nature of learners, learning, and learning spaces. In doing so, the study adopts the interpretive phenomenological approach to document and makes sense of the perceptions and experiences of learners caught in the shift.

## Research questions

Emerging from this attempt to make sense of the phenomenon of multiple spatial shifts, the present study seeks to explore the following research questions:

1. How has the phenomenon of multiple spatial shifts during the pandemic impacted the perception of conducive learning space?
2. How has the phenomenon of multiple spatial shifts during the pandemic impacted the nature of learners, their sense of self and others, and the process of learning?
3. What are the implications of mobility across and within various learning spaces on the future of higher education?
4. How can the mobility paradigm bring about new insights to understand learning and learning spaces in contemporary higher educational scenarios?

### Learning space and embodied cognition

Learning spaces, as pointed out before, have been a neglected point of inquiry in the existing pedagogical practices and structures (Chattaraj & Vijayaraghavan, 2021; Ellis & Goodyear, 2016). This trend can be traced back to the dominance of the rationalist approach to education that has conventionally separated cognitive knowledge from embodied knowledge (Kerka, 2002). The rationalistic approach, based on the mind–body dualism, has conceived teaching and learning to be a transmissive process where the focus is on ‘mind-training’ (Wang & Zheng, 2018) and the development of the ‘Cartesian-subject’ (Purser, 2018). However, the advent of the theory of embodied cognition has brought to focus the interconnection among the mind, the body, and the environment where the brain dynamically interacts with the body and the body is embedded in the environment and dynamically interacts with the environment (Wang & Zheng, 2018). The theory of embodied cognition thus focuses on the spatially situated and interactive nature of the learning (Carvalho & Yeoman, 2018) thereby acknowledging both socio-constructivist (Vygotsky, 1978) and socio-material (Fenwick et al., 2015; Sørensen, 2009) nature of learning. Sense, perception, mind–body action, and reaction thus become central to the process of learning thereby bringing the body and the experiences of its spatial occupancy into educational theory and practice (Cox, 2018; Hopwood & Paulson, 2012; Kerka, 2002). In this theory, learning space is thus perceived to be in interaction with the various forces that shapes and reshapes it thereby elevating the sociological aspect of space as has been understood by social theorist Lefebvre (Harouni, 2013).

The theory of embodied cognition has complexified how learning can be understood and conceptualized in a virtual or cyberspace that is non-identical to the three-dimensional physical space but is rather a mere stimulation of the real space (Galik & Tolnaiova, 2020). The limited literature available in this area points out that cyberspace is often conceptualized to be a non-space where the learners and the instructors experience a sense of disembodiment (Dreyfus, 2008; Pacheco, 2020). Further, a handful of studies have argued that there is an extension of embodied selves of the learners, the instructors, and the peers in cyberspace where they face complications and disruptions in the perceived and habitual practices of teaching and learning (Kazan, 2007; Land, 2013). The experiences of disembodiment or extended embodiment are not much dealt with in the literature related to virtual and hybrid learning spaces that require plenty of spatial adjustments to engage in the process of learning (Goodyear, 2020; Zydney et al., 2019).

### Classroom actors and learning

The theory of embodied cognition necessitates the need to understand the roles of the classroom actors i.e. learner, instructor, and peers in the process of learning. The transition of the conventional instructor from being the center of the formal teaching–learning process to a mere facilitator is widely acknowledged in literature (Kahl & Venette, 2010). Lee and Tan (2018) argue that in a digitalized-global world, the role of the teacher or instructor is that of a ‘pedagogical-weaver’ who in the classroom weaves the bits and pieces of knowledge provided by the learners. The easy access to knowledge owing to digitality has fostered collaborative peer learning, and the establishment of online and blended learning models have furthered the practice (Altınay, 2017; Peters & Romero, 2019). Studies show that peer learning leads to the reduction of anxiety and enhancement of motivation among the learners (Cropp, 2017) and enables them to engage in cyclical interactions thereby promoting self-directed learning (Zimmerman, 2008). Peer learning gained further prominence with online and blended learning modes (Raymond et al., 2016) that necessitated the learners to engage in self-regulated learning (SRL) (Garip et al., 2020; Stephen & Rockinson-Szapkiw, 2021; Zimmerman & Schunk, 2011). Studies show that SRL promotes peer interaction and enhances learners’ satisfaction in online and blended learning contexts (Lim et al., 2020). SRL is based on the social cognitive theory that perceives learning to be relational, based on the interactions between the learner (self), their behavior, and the learning environment. Any change in one of these three factors impacts the other factors thereby impacting the overall experience of learning (Schunk et al. 2008 as cited in Lim et al., 2020).

### Philosophical and theoretical underpinning

The study draws insights from the works of Andre Lefebvre and Maurice Merleau-Ponty to understand space. Beyond the conventional notion of space as a setting, a concrete object, and a container, the study approaches spaces as relational (Merleau-Ponty, 1962), socially constructed, and lived through experiences and practices (Lefebvre, 1991). The argument that space and spatial practices are being simultaneously structured and defined by each other (Cresswell, 2004) is significant to locate the learning spaces and practices in the context of the pandemic. The mobility paradigm emerging within the purview of spatial studies adds a further dimension to learning spaces in this context. According to Urry (2007), five modes of mobilities and their complex interconnectedness make social life, and spatial practices possible (Sheller, 2017). These five modes of mobility involve 1. corporeal travel like the movement induced in the offline context organized in contrasting time and space patterns, 2. physical movement of objects to consumers and people, 3. imaginative travel through images of places and people traversing print, and social media, 4. virtual travel that transcends space, distance, and time through digital mediums, and, 5. communicative travel that involves person-to-person messages via text, smartphones, and other similar technologies.

The phenomenological model of embodied skill acquisition is insightful here to locate and make sense of the changes brought about by mobility within and across learning spaces. Zhu (2018) refers to the five stages of embodied skill acquisition inspired by Dreyfus and Dreyfus’s (1999) work. In the first or *novice stage*, the learners are without experience and try to remember the rules and priorities of a context and situate it within the new context. In the second or *advanced beginner stage*, the learner begins to develop the basic understanding of performing in the new context that can be further supported by scaffolding activities. A learner in the third or *competent performance stage*, having gained considerable experience, begins to cope and perform appropriately in a vast number of very different situations. In the fourth or *proficient performer stage*, given an elaborate experience and history of exposure to varied situations, the learner develops an intuitive behavior and does not anymore rely on reasoned responses. In the final or *expert performer stage*, the learner displays an immediate intuitive response to the context and exhibits the properties of an experienced expert. The embodied model of skill acquisition, grounded in phenomenology, posits that a form of intuition rooted in the lived experiences leads to the expertise of the learner over a socially co-created situation that can not be reduced to the act of rule-following (Purser, 2018).

The lived experiences and the interpretations of these experiences are an essential part of making sense of *being-in-the-world* in the interpretative phenomenological approach (Horrigan-Kelly et al., 2016). Grounded in the works of Heidegger (1962), IPA foregrounds the possibility of interpretation of any phenomenon. For Heidegger, the essence of *being-in-the-world* is relationally and reciprocally constructed through the interactions among the self, the others, and the objects. This making-sense of *being-in-the-world* is primarily informed by a critical process of interpretation that enables understanding of every encounter. Further, Meleau-Ponty’s (1962) observation foregrounds that space and the experience of embodiment within these spaces become essential to this process of interpretation. Therefore, the phenomenon of spatial shift can significantly influence how learners interpret and make sense of the learning space and learning in the contemporary educational scenario.

## Methodology

### Context of the study

The study was conducted in a Southern Indian University that has campuses across India and caters to the higher educational requirements of more than 27,000 students every academic year. As a response measure to the challenges posed by the pandemic COVID-19, the university shifted to Emergency Remote Learning (ERL) (Chattaraj & Vijayaraghavan, 2021; Hodges et al., 2020; Schultz & DeMers, 2020) by adopting a complete-online mode of instruction in the fall semester of 2020. The complete-online classes involved both synchronous and asynchronous modes whereby the learners were expected to join in real-time for contact classes in the synchronous class hours allocated on their timetable and had to engage in individual/peer-group activities assigned by their instructors for their asynchronous learning requirements. The university used various Learning Management Systems (LMS) and video conferencing platforms like Microsoft Teams, Cisco WebEx, and Google Suite. Beside the platforms provided by the university, the learners also initiated peer interactions by forming learning communities and communicating through various social media platforms. With the reduction in the number of COVID-19 cases, the university opted for a hybrid learning mode in the spring semester of 2021 where the learners were provided an option to join classes either online or in physical classrooms. The instructors were mandatorily present in the physical classroom spaces. Similar to the complete-online mode, LMS and video conferencing platforms were primarily used to engage learners joining classes online and the practice of synchronous and asynchronous hours continued in the hybrid mode as well.

### Study design

As the centrality of the perceptions and experiences, past and present, of occupying various spaces and their impact on learning form the core of the present study, it adopts a qualitative methodology to understand and interpret the learners’ experiences and perceptions. More specifically, the study utilizes the interpretive phenomenological approach (IPA) that is located within the lived experiences and structured consciousness of humans (McGaha & D’Urso, 2019) and is credited with uncovering phenomena by unraveling the layers of forgetfulness or hiddenness present in the everyday existence (Frechette et al., 2020). IPA is grounded in phenomenology, symbolic interactionism, hermeneutics, and ideography (Smith & Shinebourne, 2012). Owing to the nature of the study, the questions used in the semi-structured interviews were kept open-ended and non-directive and were constructed, reviewed, and thoroughly revised (Moustakas, 2011). The general questions used in the interviews are presented in “Appendix 1”.

### Recruitment

The study was granted ethical approval by the Ethics Review Board of the host university. First-year undergraduate and postgraduate students, who were on the verge of completing two academic semesters during the pandemic COVID-19, participated in the study. These students had joined online classes in the ERL mode in their first semester of college and experienced a hybrid mode of learning in the second semester. The study invited participants from first-year programs as it was anticipated that their university learning experience would be unique as they were all freshmen and had no prior experiences of occupying the present university space with their peers and instructors. The uniqueness of their situation was further enhanced by their experiences of learning across a varied range of spatial shifts brought upon by the pandemic. Attempts were made to include a representative number of students from both the undergraduate and the postgraduate levels as it was predicted that the way learning space was approached, experienced, and utilized would be influenced by the prior experiences of higher education and disciplinary orientation of the students (Meyer & Land, 2005). Further, attempts were made to ensure that a representative number of students joining hybrid classes in the online mode and offline mode was maintained.

### Participants

Six first-year undergraduate students majoring in English Studies and three first-year postgraduate students mastering in English and Cultural Studies volunteered to participate in the study. A total of nine participants were considered ideal for the study as phenomenological studies are expected to have a sample size of four to ten participants (Mastel-Smith & Stanley-Hermanns, 2012). By the ninth interview, a significant insight into the lived experiences of a fairly homogeneous sample of learners was gathered, and more importantly, both the investigators felt that the experiences reported in the interviews were getting repeated. Thus, the investigators felt that a saturation of data has been reached, and there on decided to deeply examine each individual case based on the tenets of the IPA methodology (Garip et al., 2020). The demographic characteristics of the participants are provided in “Appendix 2”. Information about the level of study, age, gender, religious affiliations, and whether the learner had prior experience of ERL, hybrid learning, and joined hybrid classes online or offline is provided. To maintain the clause of anonymity and protect the participant’s identities, the names were replaced with pseudonyms during the process of transcription by the first author.

### Data collection

Pre-interview questionnaires comprising questions on the learners’ profile and the informed consent clause were emailed to the participants. Post the participants’ response, a mutually convenient time was decided for the individual, in-depth interview where both the research investigators and the individual participants were present. The interviews were conducted and recorded over Google Meet in March and April 2021. The average length of the interviews was 26:01 min. The verbatim transcriptions of the interviews were made before being explicated for the study.

### Data explication

The explication of responses for the present study is inspired by Colaizzi’s strategy (Shosha, 2012; Tuffour, 2017). The interview transcripts were (1) read and re-read several times, (2) notes and comments made individually by the investigators were discussed together, (3) dominant patterns emerging from the interviews were noted and probed further to formulate the meanings, (4) the formulated meanings were sorted into categories, cluster of themes, and sub-themes, (5) attempts were made to establish an interrelationship among the emerging themes, (6) attempts were made to describe and evaluate the fundamentals of the phenomenon in question, and (7) attempts were made to keep the meaning and essence of the recorded experiences.

### Study rigor

The principles of sensitivity to context, commitment and rigor, transparency and coherence, and impact and importance were adhered throughout the study process to add rigor to the study and address the concerns of validity and reliability (Yardley 2000 as cited in Garip et al., 2020). The criteria of trustworthiness were promoted and maintained in the study by adhering to the notions of credibility, dependability, transferability, and confirmability (Long & Johnson, 2000; Villa et al., 2018). Trustworthiness was ensured through a (1) prolonged engagement of the researchers with the study, (2) conducting multiple interviews and making notes, (3) preserving the confidentiality and privacy of the participants, and (4) by member checking and triangulation (Villa et al., 2018; Vogl et al., 2019). The research investigators ensured that they were responsive, adaptive to the situation, and sensitive to the participants. The investigators ensured that the questions were rigorously probed and the responses paraphrased in real-time to ensure validity and reliability of the study (Murray & Holmes, 2014). Furthermore, IPA being a double-hermeneutic process, reliability was ensured in this study by building consensus between the researchers through exhaustive discussions and deliberations over a period of time (Rodham et al., 2015). Sensitivity towards context was maintained throughout the study and can be observed in the verbatim quotes from the participants provided in the result section of this study. This not only ensured that the participants’ voices were well represented but also enables the readers to trace the interpretations and findings of the study (Spiers et al., 2016).

The strategy of Comprehensive Triangulation by Denzin was adopted for the study (Flick, 2018). This ensured that the study contained investigators’ triangulation whereby both the researchers in this study not only collaborated in the process of analysis but were also present during the interviews to minimize biases coming from an individual researcher. Theoretical triangulation was ensured by incorporating theories from the fields of sociology, psychology, and education to understand the complex phenomenon of multiple spatial shifts. Methodological triangulation within the method was ensured through conducting interviews using the IPA approach and analysing the conversations. These triangulation strategies adopted in the study further ensured a systematic triangulation of learners’ perspectives.

## Results

The interviews revealed that the phenomenon of multiple spatial shifts that occurred in the span of one year of the pandemic COVID-19 has significantly altered how learners perceive and experience learning. Some of the dominant patterns emerging from the experiences of the various spatial shifts are (1) the changing perception of conducive learning space, (2) the changing perceptions and roles of the various classroom actors, and (3) the evolving nature of the learners and the learning process. These patterns are further discussed below.

### The changing perception of conducive learning space

Given the experiences of learning across various spaces—physical, online, and hybrid—the participants’ accounts reveal that their perceptions of the various spaces were constructed through a relational experience of learning across these spaces. The accounts documented are discussed below.

#### Experiences of offline classroom space

The mobility of the learners across various spaces has brought in a heightened sense of value, largely based on the memory of corporeal and visual learning experiences in the conventional, offline classroom space as is reflected in the following accounts.

I love classrooms because in a classroom you have a lot of friends that you can interact with, you learn from them, and they learn from you, and then with professors also like it's much more easier to raise your hand physically in class... if you don't have a good internet connection then you might miss out on something. That is not going to happen in a physical class and 100% concentration is there. (Aditi)

...if I had come offline, then I would get more face-to-face interaction, I could see how my teachers are responding to my answers. And I could also get a hands-on experience of what they have to offer me in return for my answer. If it is wrong, then they can properly correct me. It happens in the online scenario also, but I think that their facial expressions and their smile is very important when I'm answering something. (Nigel)

When we are in online classes.., we are always conscious about what we are speaking, it's almost like someone is watching us, hearing it, and anybody can cut any of that out of the context and replace that in an incorrect manner. Whereas in class (offline), it's more of a dialogue.. and I can exactly look at my classmate as well when she's speaking, and I can say the same about my instructor. So there's much more expression involved, there's more dialogue involved, and it's more organic. (Stuti)

#### Experiences of online learning space

The perceptions of online learning space are constructed in relation to the memories and experiences of the offline classroom space. Some of the positive aspects of the online learning space as opposed to the offline classroom space as reflected in the following accounts are freedom, flexibility, and adaptability.

The most positive aspect I find about this online thing is that we do have the freedom, a certain level of freedom to engage with the classes. For example, in my case, I do feel a little restless in classes. So, when I am in an online scenario, I feel I am less monitored and that gives me a sense of freedom. (Nigel)

Even after starting the online classes I didn't have this feeling of missing out on offline classes, and then after a while, it was a broad interaction, really. I was getting used to online. (Vahin)

..in a way my adaptability has increased, so now I can confidently say that I can pursue education and I can pursue learning, not only in a physical setup but in an online setup. (Adya)

I always have music playing in the background, music helps me focus even in online classes. There's light music playing in the background it is easy to focus, and I can't do that in (offline) classes and at the same time, when there's a particular content being discussed in class and I have a slight difficulty in understanding, what I do is I can immediately take my phone and Google it and search a little extra on it. (Rohan)

#### Immediate sense of alienation in the offline classroom space

Post the experiences of learning online for a span of more than one semester, interestingly, some of the participants also reported an immediate sense of alienation with the physical classroom space, as is observed in the following accounts.

So even if we have a normal class as in my 12th grade.. extremely conventional with a board and teacher, still the gap that we had for a year has changed me as a person, to an extent… sitting home for one year, your habits change, the way you see schooling changes, and when you come to offline classes, you just feel you're starting from ground zero again. So yeah, that feels a little alien but I don't feel that in a factual sense there is much difference. (Rohan)

...during online we get used to not doing that (coming to campus every day) and when you come in here, you have to be stricter. You have to be more disciplined... I wouldn't consider that challenge, but coming from an online platform it's scary. It kind of is. (Vahin)

Here, the participants’ accounts reveal that the perception of the learning space is relationally constructed through the memory and comparison of experiences across online space induced by the pandemic and the return to the offline formal classroom space. The absoluteness associated with the offline classroom space as the sole conducive learning space seems to gradually evolve.

#### Experiences of hybrid learning space

There is a general sense of preference towards complete-offline space and complete-online space as compared to the hybrid learning space. The accounts reveal that the hybrid space is perceived through a sense of being ‘in-between’.

To be honest, the first thing that comes to my mind when I think about hybrid is Homi Bhaba’s concept of Hybrid Theory. It's like how everything it's, it's a gray matter. It's not black and white... I did not join in the initial days in the offline hybrid structure…(Here) We do not know what is happening because we can hear muffled voices. So, it's almost like we (online learners in hybrid mode) are on a threshold. We are neither in nor out, but we're just a participant without a voice… So, I would say, one should only stick to either online mode or offline mode, given the situations or privileges, but hybrid is a complete failure. (Stuti)

...I think we (online learners in hybrid mode) are more in a virtual space and they (offline learners in hybrid mode) are in a real space - my peers who are in the class, they are at a much better advantage, I believe, than we are in a virtual space. (Nigel)

No, it doesn't completely feel offline ...you still have to join the class online. We (offline learners in hybrid mode) have to unmute ourselves sometimes and talk to the computer also because our classmates have to hear us, and all of our classmates are not in class. So, yeah, it's totally different. (Vahin)

The students who are there on campus (offline-hybrid) are more advantaged because we have immediate responses. But then when it's like online students (online-hybrid) have to contribute it's a several processes for them to wait for our (offline learners in hybrid mode) turn to get over. And then if they want to say anything spontaneously, it's not possible for them. So I think as a class overall, it should either be completely online or completely offline... with respect to responses and everything, I do feel that it's kind of delayed for the people who are online (online-hybrid). Yeah. (Savi)

#### A heightened sense of neglect in the hybrid mode

The sense of being ‘in-between’ that characterizes the experience of the hybrid mode is further heightened by the sense of neglect experienced by the learners attending the hybrid mode via online, as reflected in the following accounts.

And once the hybrid mode started, since most of us felt a little neglected while attending online (online-hybrid) - we felt that we were less accountable… then we felt that maybe we should just attend offline (offline-hybrid) classes for better understanding. (Rohan)

I think this one (hybrid) is a little more difficult than the overall online. I think it's a little more difficult for the online (online-hybrid) students, because at times during class discussions, maybe the professor is a little bit distant from the microphone so we are not able to follow the discussion, clearly, or maybe the students (peers in offline-hybrid) are not audible, so we're not able to hear what they are saying.. (Adya)

Some of the significant observations emerging from the accounts are (1) the perception of conducive learning space is relationally constructed through the experiences of learning, occupancy, and performances across various learning spaces, and (2) there is also a general preference towards a complete-online or a complete-offline mode of learning than a hybrid mode. Most often, this dissatisfaction in hybrid mode largely emerges from the experience of being in neither the online nor the offline spaces completely. Further, there is a gradual acceptance towards complete-online learning mode thereby the absoluteness associated with the conventional built classroom space as the sole conducive space is changing.

### Changing perceptions of actors across learning space

The learners’ perception of conducive learning space is largely shaped by the presence of certain actors based on the notion of the conventional built classroom spaces. The multiple spatial shifts however have impacted the mode in which these actors and learning spaces are perceived.

#### Lack of corporeal presence of classroom actors

The accounts reveal a larger sense of freedom in the online mode mostly associated with the lack of corporeal presence of various actors, specifically the instructor who is conventionally understood to be in charge of the learning process. However, the sense of freedom is also coupled with a sense of intimidation, and alienation-exhilaration that arise from experiences of interactions enabled by technological mediums. These changing experiences of learning and the perception of learning spaces, and actors, informed by the spatial shifts, can be observed in the accounts below.

We do have a certain level of freedom to engage with the classes (online). For example, in my case, I do feel a little restless in classes. So, when I am in an online scenario, I feel I am less monitored and that gives me a sense of freedom. (Nigel)

It was intimidating... I cannot see my classmates as such, I do not know, on what level everyone is… it was really scary because people ask such questions, it's jargon and everything, and it was intimidating. (Savi)

When I'm answering to a computer, as we are doing right now, it feels very elevated and alienated, so like we are actually talking to a real person, but at the same time it feels, I'm talking to a computer which is not actually alive… It's extremely alienating... In online class there is less accountability, it just becomes like a YouTube mode, like what we call 'a good content' to look at. (Rohan)

… it used to be just professors and there was a screen and there were some students, there was no direction at least. I don't know if this is the right term to use but it was lacking a little bit of familiarity. (Babik)

The mixed sense of ‘freedom’, ‘elevation’, ‘non-familiarity', ‘intimidation’, and ‘alienation’ expressed by the participants are dominantly shaped by the experiences of disembodiment of the ‘self’ and the ‘other’ actors in the online spaces. However, the learners also mention certain strategies adopted by them to cope with these challenges. These strategies are discussed as the next dominant pattern (pattern 3) in the paper. Despite the challenges, the accounts reveal a greater sense of acceptance of the complete-online mode as compared to that of the hybrid mode.

#### The sense of being in-between in the hybrid mode

The experiences of hybrid learning are fraught with challenges in terms of equal access to synchronous classes by online and offline learners. The online learners attending the hybrid mode reported an experience of neglect informed by a sense of (1) not being a ‘real’ or ‘actual’ student, (2) being concurrently present in two different spaces, and (3) disinterest and withdrawal from participation in learning activities. Some of the accounts that reflect these observations are presented below.

And once the hybrid mode started, since most of us felt a little neglected in the online experience (online-hybrid) like we were less accountable… We felt that maybe we should just attend offline classes (offline-hybrid) for better understanding… I just felt that maybe teachers felt comfortable interacting with the actual students over there, and the questions that came to online students were very less, and we felt a little bit late, lagging behind of what we were doing, and the response. (Rohan)

Ma'am, I'm virtually present in the online space (online-hybrid) but I think I'm also accommodated in the offline space (offline-hybrid) when the teacher asks a question and I need to answer so people who are in the offline setup (offline-hybrid) are also able to listen to me. So there is a presence in both the online and offline space. (Adya)

..we (online-hybrid) are largely missing out from sources because when they are offline (offline-hybrid), we do not get that kind of interaction with them... They (the peers in offline-hybrid classes) do forget to unmute themselves, and we can only hear that from the teachers’ microphone, so that becomes very difficult, as I consider my peers very valuable because they are actually providing good answers and good insights to certain important factors about the class. (Nigel)

..when everyone was online (complete-online mode) so people were participating more. But now, like when half the students are offline (offline-hybrid), and half are online (online-hybrid), so the online (online-hybrid) ones are basically slacking off.. because now the students are offline (offline-hybrid), so they will participate. But I don't think that's good for our learning. (Aditi)

The sense of in-betweenness and disembodiment become the dominant challenges related to the technologically-enhanced hybrid learning spaces. While the sense of disembodiment associated with various actors in the online space is a persistent trait, the learners also associate a sense of freedom and flexibility emerging from the lack of corporeal presence of the classroom actors, especially instructors. However, the hybrid space seems to adversely impact the learning process considering the inequitable access to resources and actors in synchronous learning. The changing perception of learning space and actors induced by the shift also seems to have impacted the existing learning practices as is discussed in the next pattern.

### Evolving nature of the learners and the learning process

The nature of contemporary learners is significantly shaped by the constant interactions with digital technologies. However, the phenomenon of multiple spatial shifts across various learning spaces has made this interaction with digital technologies an integral part of the learning context and process.

#### A gradual sense of comfort in the online learning space

A general sense of comfort towards online learning spaces can be observed in the accounts provided below.

..what if my internet cuts down in the middle of my attendance process/class. So there were some fears like that. And then it became a common practice. It got wired into our brains and it started running smoothly. That's it. (Nigel)

The good thing about online classes was that it was extremely flexible and independent, there are some characteristics that students themselves got adapted to like when we are in a classroom (complete-offline mode), there are certain etiquettes we have to follow, and it's not necessary that all the etiquettes make us feel comfortable. Sometimes a little music in the background feels comfortable to study, and when you're hungry you can grab a little snack now and then. But that cannot be done in a classroom situation. That freedom was like a fair amount and I like it. So I prefer online classes and I would be rather comfortable learning that way. (Rohan)

Definitely, it has created an impact, I think both positive and negative; negative in the sense that you are adapting to the changing surroundings in a very short period of time, and positive in a way that my adaptability has increased, so now I can confidently say that I can pursue education and learning not only in a physical setup but in an online setup also. (Adya)

The sense of comfort noted in the accounts emerges from a sense of familiarity of learning in a complete-online space for an entire semester. Furthermore, this sense of comfort can also be traced back to the adaptable nature of learners to new learning spaces, specifically the complete-online learning mode.

#### Self-aware learners and self-directed learning

The self-awareness displayed by the learners can be traced to awareness of (1) the new skills they have acquired in the process, (2) the enhanced proficiency in accessing online learning resources, and (3) their process of learning, as reflected in the accounts below.

So, one of the very interesting habits that all of us have acquired, not all of us but like some of us have acquired, is that, like, when we are in lectures we take notes, usually. But if those lectures are recorded, we can go back to them and get notes, again, or we can go back to them and understand them again… And also, when it was online, it was a lot of internet-based, And not a lot of article-based, not the concept of book and pen… Online mode is really technologically advanced… (pre-pandemic) I used to just study in an enclosed room, just me. I used to read out stuff, and as I said, I repeated stuff aloud. But in the online mode, it was easy to interact with people, and so it was new for me to get more opinions and interpretations of the texts that we are reading. So it was like a collaboration of a lot of minds. (Babik)

I've learned to use all these different platforms and different libraries, I didn't even know that there were different libraries existing. So I accessed a lot of articles... I know how much time I ideally take to read which I did not know before (pre-pandemic) because I used to just do it as per the text but now, I know that okay, I at least need like 2 hours to read it twice, and then I need two-three hours for extra research. So I figured that out. (Savi)

It was necessary to have peers in the online mode… A student can learn individually in offline mode, but he cannot learn online individually so groups are kind of necessary. (Deep)

When it was online I felt that it was more necessary that I interacted with more people in class. Offline you go to a group, you fit yourself into that group, and all you do is you talk to them. But in the online mode because of the ease of communication or whatever you don't have to approach them face to face. For some reason, the engagement was more, I mean I talked to a lot of people but it’s different from making friends, but still I had to interact with a lot of people when it comes to learning a subject, or how they understood it and all. (Vahin)

The accounts reveal that there is a significant shift towards self-directed learning. While the learners display a great deal of awareness about their learning process, they also demonstrate an enhanced awareness about the role and contribution of their peers in their learning process.

#### Learner-created knowledge-based communities

The experience of pandemic and the various shifts emerging within the context has resulted in an enhanced need for peer learning and a conscious effort from the learners to build communities that become a common platform to share anxieties, and support, motivate, and help each other academically and otherwise, as reflected in the accounts.

It's all because of the difficulties that have been imposed on us by the pandemic, individualistic learning was more possible during pre-pandemic times but now that we are all imposed by challenges, the only source of help that we have is each other. We rely on each other, take each other's support and help to get through these times. (Adya)

If we start our first day as an offline student, it is much different from the friendships that I made as an online student, because as everyone is feeling the heat of this pandemic and this online education more, every student, every one of my friends is actually ready to give any kind of help, and I am also willing to give any kind of help that might be. So I believe that there is a very strong bond that has been virtually made over this network in this online scenario… People are more like yeah you text me if anything you need. There is a friendship since everyone is equal in an online scenario. We do feel that she is having a problem, he's having a problem, and I am having a problem. So I am easily able to empathize with my entire class. (Nigel).

During this assignment, one of my group-mates was not really comfortable, even to unmute herself because every time she unmuted, there were some background noises that involved her family, and she was quite embarrassed by it. So we told her “you can keep yourself mute, and you can only type in the comment section if you have to”, and that actually gave her a lot of space to be confident, and finally there came a time when she was comfortable unmuting herself and being a participant. (Stuti)

The accounts display that within the online mode, there is a natural tendency among the participants to depend on their peers as opposed to that of the offline mode. If the conventional offline space enhanced interactions with peers primarily based on personal equations, the online space seems to promote interactions and community building based on knowledge sharing and acquisition. Further, the challenges imposed by the pandemic also become a means to build a community based on the values of empathy, compassion, and shared needs of learning, emerging from the context of being in a crisis together.

#### Learners’ perception of the future of higher education

Emerging from the experiences of mobility across and within various learning spaces—physical, online, and hybrid—the learners also report a certain level of awareness of the changes in the domain of higher education. Some of the accounts below reflect these changes noted by the learners.

..so we lose, we are very well losing that factor so the pen and paper goes out, and the device sets in as an agent for communication, you are not going to give verbally, everything in a classroom so probably a WhatsApp text or a Google Classroom announcement is what people are looking for. The problem is that in five to ten years we probably will lose the offline classes method because online seems convenient because we see platforms (edtech) coming up and they're rather successful so there are people who actually prefer it, and they got a very big hype during the pandemic so people are getting comfortable with it. And there's a probability that this on-campus structure is going down. (Rohan)

It’s possible I think a lot of the courses had used these modes (complete online/hybrid), even before the pandemic. If I'm not wrong. I don't know how popular it was because if I had an option before the pandemic to do an online course, I would think twice before doing it, and I would prefer going offline. So I'm not sure how popular it was but maybe the popularity might rise after the pandemic. (Vahin)

I'm afraid that it will be the future mode of education. I don't like it personally. But then there is a chance that after institutions find it more easy to manage and more economically feasible, they might actually prefer hybrid classes or even online classes. And for those people who actually find, for example, my parents. They do tell me that, oh, this online class has been very beneficial to our family because if you would have gone to Bangalore, back in July 2020, it would have set us back at least a lakh rupees on rent. So, people who are from a difficult economic situation, they will definitely find such online classes helpful for them. So if universities would find that it is economically feasible to them also, then there is a high possibility that hybrid and online classes could become the future of education. (Nigel)

Ma'am, if it is (hybrid/online as future), I don't think it will have a positive impact, if it shapes the education system, especially in a country like India where we have to take care of a lot of facilities and resources at the same time when we are thinking about a learning space. And the entire idea of gender also plays an important role when we think about access to education, because not everybody will get access/choice to come to a college, and read, or get access to a mobile phone/laptop... I think a majority in our society will be deprived…the number of girls who go to schools or colleges will definitely reduce because we do understand the gender bias in our society. (Stuti)

The accounts reveal a certain sense of awareness among the learners about the implications of a shift to online/hybrid mode on the future of education. There is a general sense of acknowledgment that the mobility across and within various learning spaces has opened up the possibility of higher educational institutes to explore new learning spaces besides the conventionally built classroom spaces. However, it is important to note that there are mixed feelings about the implications of these shifts. While a few of the learners display an acceptance towards learning in both offline and online modes, and also note the economic benefits for both educational institutions and their stakeholders, they are also conscious to point out the gender and social disparities that such a shift entails.

## Discussions

The patterns emerging from the phenomenological study reveal that the phenomenon of spatial shifts has informed the perception of conducive learning space, the roles of classroom actors, the nature of learners, and the process of learning. If conventionally, the built classroom space was imagined as the desirable space of learning (Ellis & Goodyear, 2016), the movement across spaces, especially online, has complexified this perception. Some of the participants who associated a sense of ‘alienation, and ‘scariness’ with the offline built classroom spaces, post the experience of learning in the online space for more than one semester, provide insights into this complexity. The gradual change in perception of conducive learning space noted here emerges from a relational construction of the learning space owing to the experiences of learning across various spaces—physical, online, and hybrid—induced by the shifts (Cresswell, 2004; Merleu-Ponty, 1962; Urry, 2007).

**Fig. 1:**
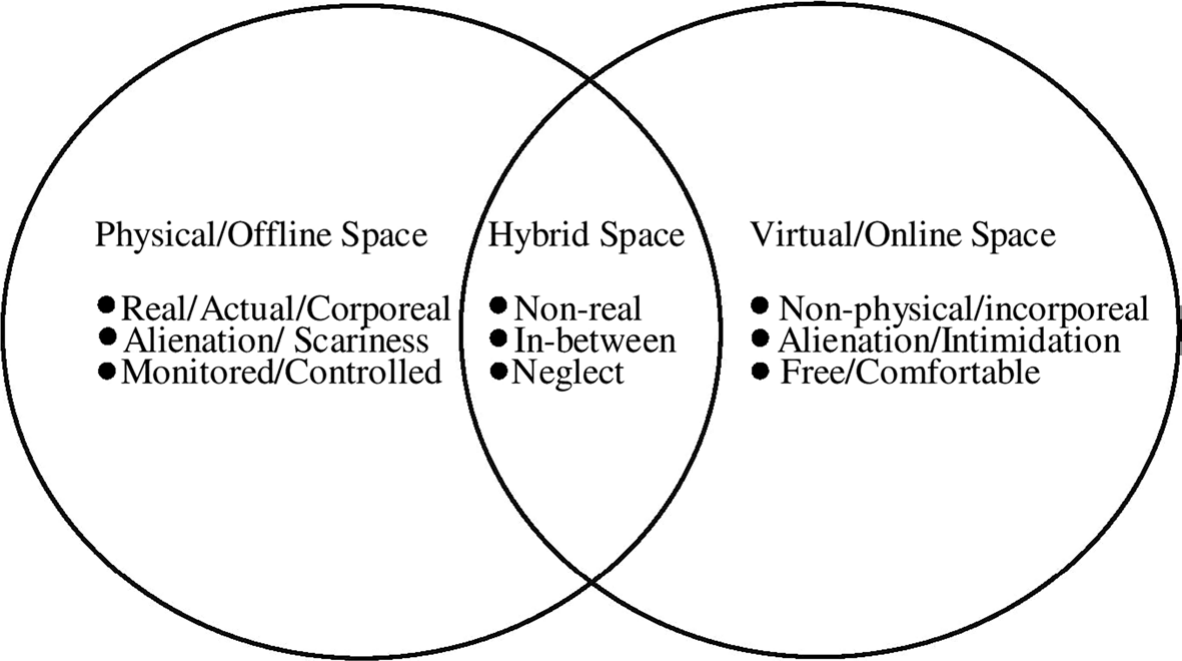
The experiences of the learning spaces

The experiences and spatial practices associated with the spaces, as pointed out in Fig. 1, also shape the notion and perception of these learning spaces. If the offline space is perceived as embodied and monitored, the technology-enabled space is shaped by a sense of disembodiment and freedom marked by the corporeal absence of the self and the classroom actors. The absence of corporeality associated with various actors is further complexified by the experiences of mobility within the technology-enabled spaces.

**Fig. 2:**
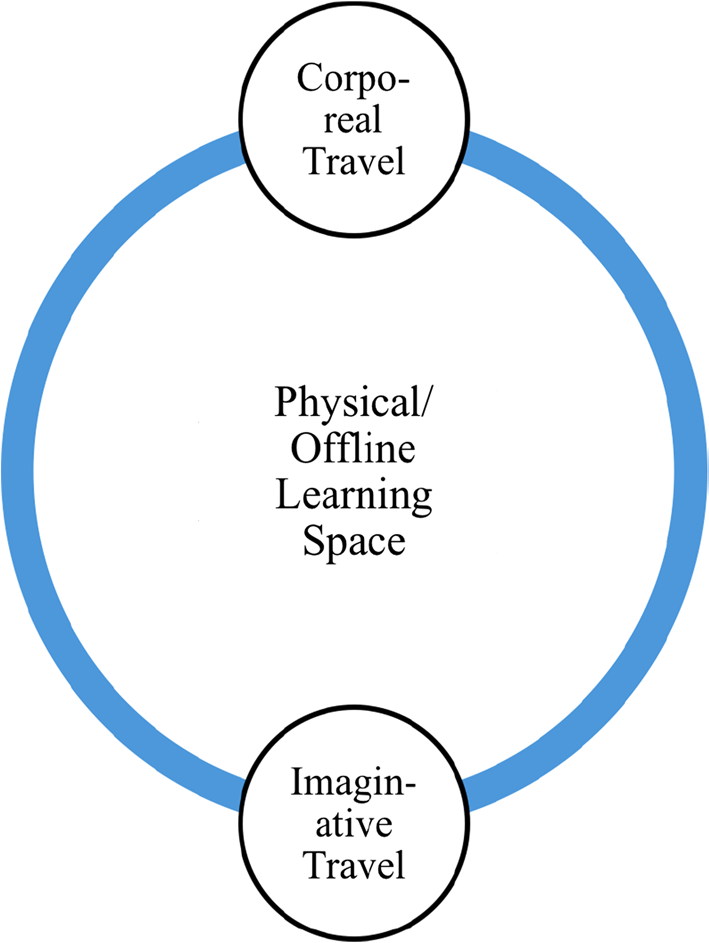
Mobility in physical learning space

**Fig. 3:**
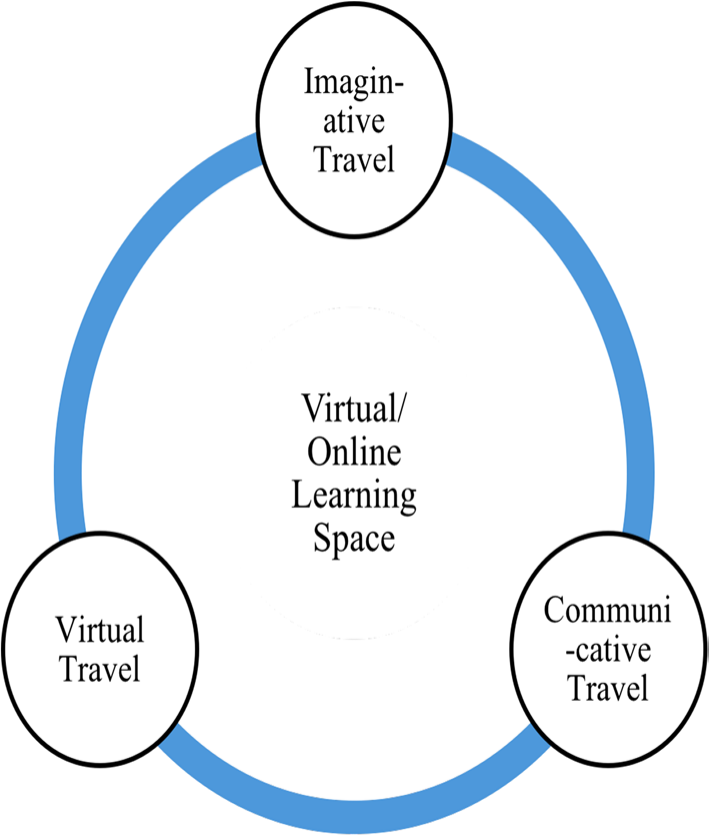
Mobility in virtual learning space

**Fig. 4:**
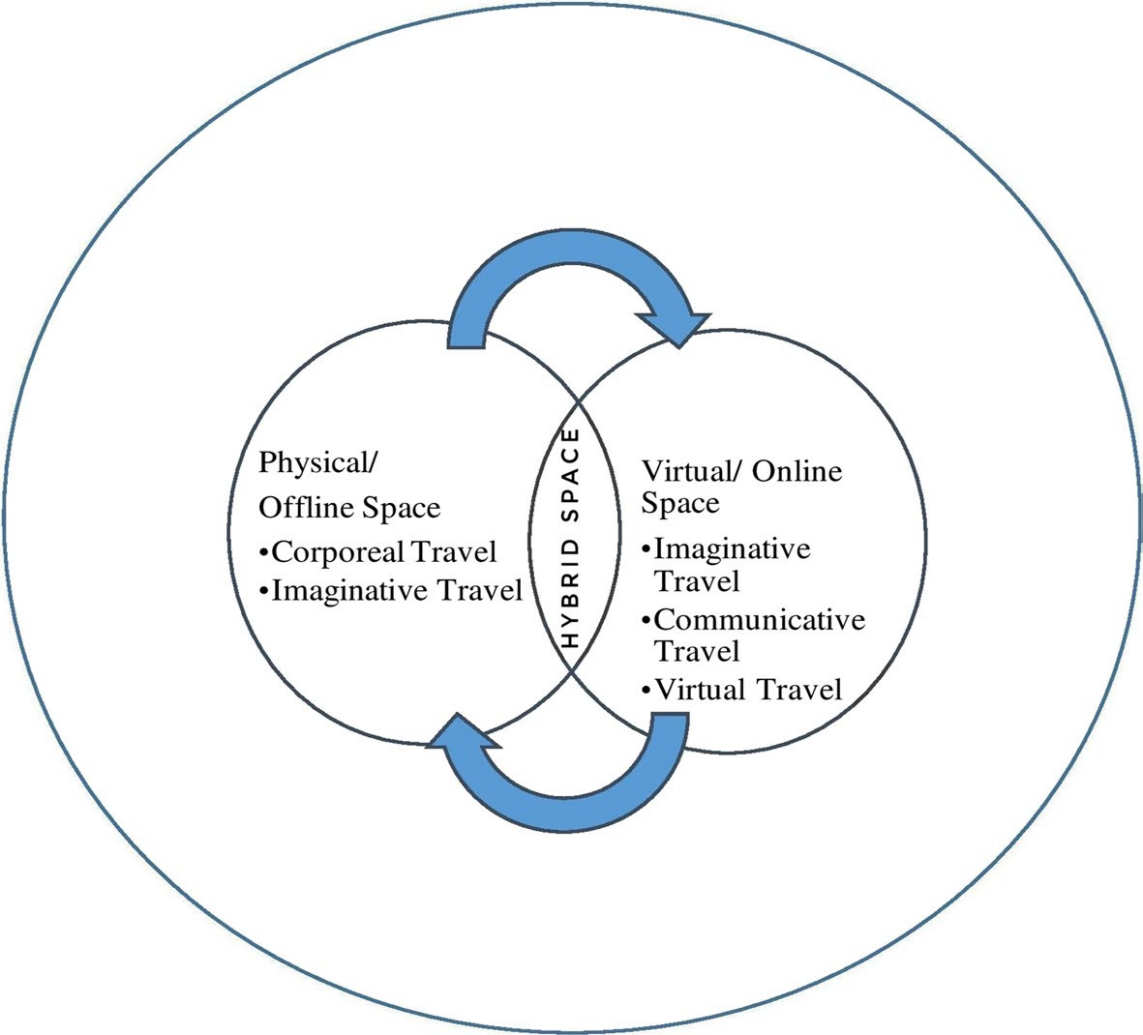
Hybrid learning space as a blended and inclusive space

As is indicated in Figs. 2 and 3., learning in the virtual/online space is enabled through imaginative, communicative, and virtual travel than the corporeal and imaginative travel associated with the offline spaces (Urry, 2007). In the technology-enabled space, like the online space, the learners negotiate imaginative, communicative, and virtual travel through changes in spatial practices. The changing spatial practices of learning include interacting with instructors and peers through technological and digital mediums, surfing through online learning resources, and the non-adherence to formal classroom etiquettes. The hybrid space that simultaneously allows all the modes of travel (Urry, 2007) is associated with a sense of neglect emerging from the corporeal presence of certain participants and the virtual presence of certain others. Further, the sense of in-betweenness associated with the hybrid space emerges from simultaneously being present in both the virtual and the physical classroom space and therefore having to adopt spatial practices that are disorienting and confusing for the learners. The hybrid learning space is imagined within the popular conviction as an inclusive and blended space that brings together the best practices of both the online and the offline mode (Deshmukh, 2021; Kaufman et al., 2021), as is represented in Fig. 4.

**Fig. 5:**
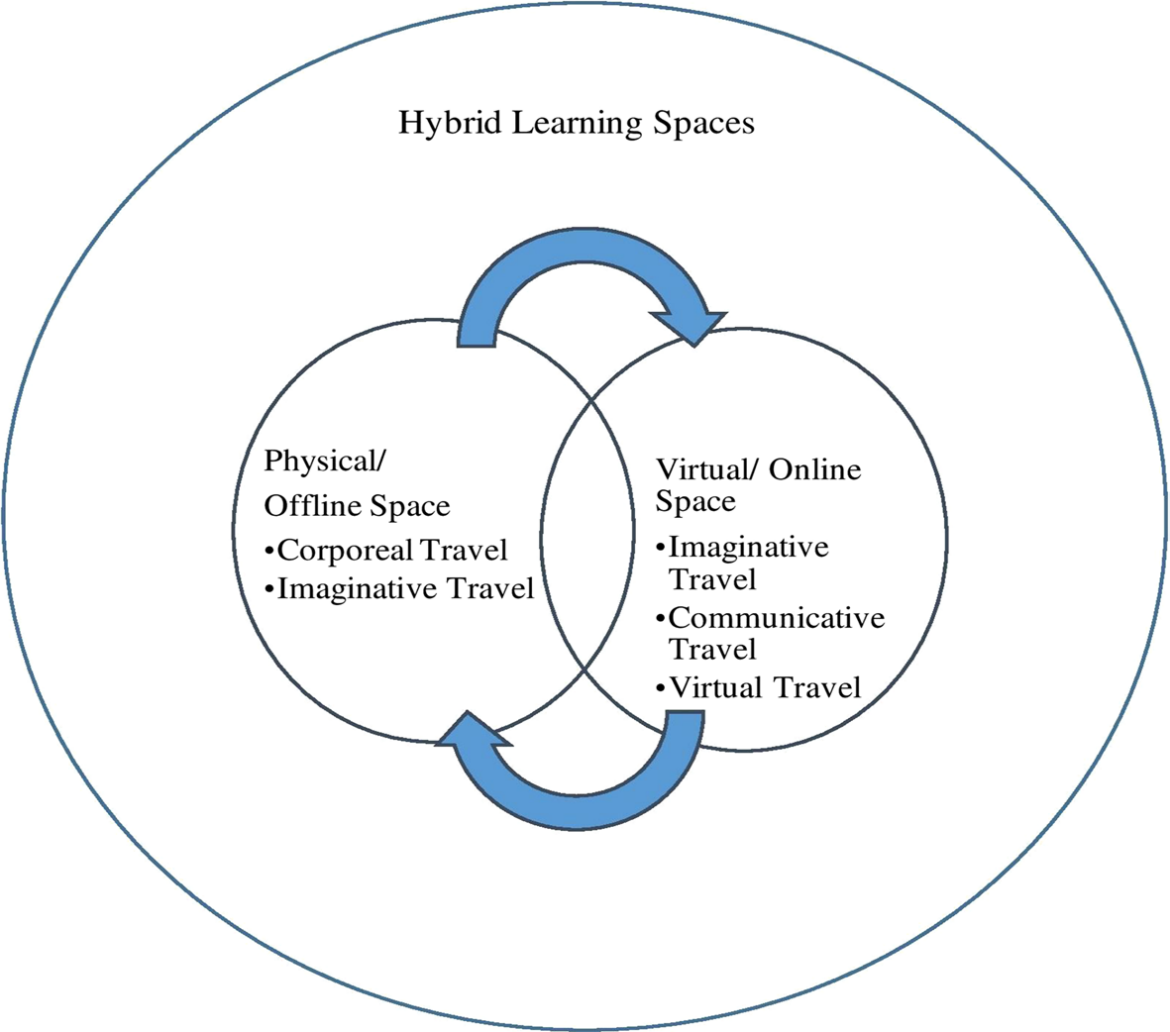
Learners’ perceptions of the hybrid learning space

However, the observations emerging from the present phenomenological study indicate that the learners experience a sense of in-betweenness in the hybrid mode. The sense of in-betweenness emerges from the sense of being concurrently present in both the offline and the online space/mode and having to adapt to specific spatial practices informed by these spaces. Such an experience, evidently, disorients and confuses the learners thereby significantly challenging the notion that the hybrid mode is a blend of the best practices as specified above. Figure 5 represents the experience of the hybrid space that is marked by ambiguity and confusion emerging from the inability to locate the self within one particular space and therefore not being able to get a concrete sense of the space and the spatial practices.

The changing nature of the learners and the evolving skills related to learning is also highly influenced by the spatial and mobility practices informed by the shifts. The conventional built spaces shaped the nature and acquisition of skills based on the embodiment of the learner within the spaces of learning (Carvalho & Yeoman, 2018; Cox, 2018; Hopwood & Paulson, 2012; Kerka, 2002; Wang & Zheng, 2018). For instance, the act of note-taking that involved an unconscious use of pen and paper was considered an integral part of learning practices in the classroom spaces. However, the shift to technology-enabled learning has resulted in the learners reporting a loss of these skills emerging from the interaction with digital mediums and the availability of recorded lectures. This can further be located in the learner-initiated changes towards self-directed learning enabled through digital literacy and knowledge-based community building (Garip et al., 2020; Lim et al., 2020; Stephen & Rockinson-Szapkiw, 2021; Zimmerman & Schunk, 2011). If the conventional built spaces were dominated by the centrality of the teacher-instructor, the online learning spaces seem to have foregrounded the significance of knowledge-based communities, peeIpl2uae#r interactions (Altınay, 2017; Peters & Romero, 2019), and the role of the instructor as a ‘pedagogical-weaver’ (Lee & Tan, 2018). One of the significant changes that the mobility paradigm has brought to focus on is the possibility to locate the learners within the stages of the embodied skill acquisition model (Purser, 2018; Zhu, 2018). Considering the changing nature of learners and their awareness of their learning process and requirements as reported in the study, the learners seem to be at the *competent performance stage* wherein the learners have gained considerable experience in the technology-enabled online learning space and are able to perform within the changing scenarios and spaces of learning (Dreyfus & Dreyfus, 1999). However, the same observation cannot be made about the shift to hybrid learning space.

The experiences of multiple spatial shifts in learning spaces, while, have made the learners conscious of their learning process, it has also made them aware of the possibilities of changes in higher education. The learners’ perception of the future of higher education aligns with the discussions and debates that indicate a transition towards a digitally enhanced higher educational system (Bonfield et al., 2020; Deshmukh, 2021). The experiences of learning across novel learning spaces have made the learners showcase a greater sense of acceptance towards complete-online mode alongside learning in the conventional built spaces. However, the learners also seem to be conscious of the digital divide that could be brought about by this imminent transition in higher education (Neuwirth et al., 2020).

## Conclusion

As technology-enabled learning becomes the new normal, investigations on spatial shifts, located within the mobility paradigm and spatial practices, become significant to make sense of the experiences of learners and the process of learning. Some of the observations that are pertinent to the field of higher education, emerging from the present phenomenological study, are 1. the change in perception associated with conventional built classroom space as the sole conducive learning space, 2. the dissatisfaction with the hybrid mode of learning and a preference towards a complete-online and complete-offline modes, and 3. a notable movement towards self-directed learning and building of knowledge-based communities through peer learning practices. The findings of the study thereby necessitate a need to revisit the way in which pedagogues and researchers have so far engaged with the various modes of learning—physical, online, and hybrid—emerging from the context of the pandemic COVID-19.

The potential offered by the spatial practices and mobility paradigm within the context of higher education is immense and can open up new inroads to make sense of learning and education in contemporary times. If the pre-pandemic learning spaces and practices were primarily dominated by corporeal travel, the pandemic-induced learning context and spaces are contingent and uneven with multiple practices of mobility and immobility. While the present study explores certain aspects of this mobility in terms of shifts across spaces and travel within the learning spaces, this paradigm seeks further in-depth engagements considering the complexities associated with mobility. Further, the experience of embodiment and disembodiment documented in this study to trace the stage of embodied skill acquisition among the learners also opens up possibilities to shape the model based on the changing nature of formal learning spaces. With the shift to online/virtual spaces, such an argument is relevant considering the fact that the technology-enabled learning spaces are shaped by the experiences of disembodiment and extended embodiment.

While the phenomenological study allows the possibility for an in-depth engagement with the phenomenon of spatial shift, the number of participants involved can be considered as a limitation. Though the nature of the topic engaged requires such an intervention, further explorations from a varied range of learning contexts are necessary to analyze and validate the patterns that emerged and the observations made in the present study. The study illuminates the potential of the mobility paradigm and argues that an in-depth engagement with mobility and space is crucial to trace the transition in the field of higher education emerging from COVID-19.

## Data Availability

All the transcripts of this qualitative, phenomenological study can be made available on request.

## Appendix 1: Questions for semi-structured interview

1. How do you perceive a classroom space?
2. What is your idea of a hybrid class?
3. Do you attend the class online or offline? Tell us why you chose what you chose.
4. How do you generally learn? Do you prefer learning alone or together with friends? How did hybrid experience impact your preferred approach to learning?
5. If online, describe your experience of attending hybrid classes.
6. If offline, where do you live? Describe your experience of hybrid class.
7. Describe your previous experience of attending online classes during pandemic.
8. How was your experience? Describe any challenges you faced and steps you took to mitigate them.
9. How is this hybrid learning experience different from the previous online learning experience?
10. How is this hybrid learning experience different from the previous offline learning experience before pandemic?
11. List a few things you like about hybrid learning and list a few of the challenges.
12. Use 4–5 adjectives to describe your hybrid learning experience.
13. Describe a day of your hybrid offline learning experience.
14. Do you feel that in the hybrid mode you occupy various spaces?
15. Do you think shifting to offline/online learning in the form of hybrid classes has impacted your overall learning experience? Why?
16. Are you happy to come back to classrooms? Is the experience of occupying classrooms the same or different than the pre-pandemic classroom experience?
17. What is it you like best about coming back to the classroom?
18. What is your idea of a good learning space?
19. Can you describe the learning spot and tell us why you chose that spot?
20. Are there any external factors that disrupt your learning experience in hybrid?
21. Can you tell us some of the things which you miss about your online learning space? Do you want to share any particular incident from your recent experiences?
22. Do you feel that now that you're back, your learning is more organized? If so, why?
23. Do you think e-learning is beneficial? What do you think are the benefits?
24. Do you think your past experiences of e-learning changed the way you learn/ participate in the learning process now that you are back on campus? If so, how?

## Appendix 2: Details of participants

**Table:**

Participant (duration of interview)	Location during the study	Age, gender and religious affiliation (through official records)	Level of study, prior-experiences of hybrid learning	ERL-experience, mode of joining hybrid classes
Stuti (31:35 min)	Bangalore, Karnataka, India	24, female, Hinduism	Masters, no	Yes, offline
Savi (25 min)	Bangalore, Karnataka, India	23, female, Catholic Chistianity	Masters, no	Yes, offline
Nigel (29:03 min)	Kannur, Kerala, India	25, female, Catholic Chistianity	Masters, no	Yes, online
Deep (18 min)	Bangalore, Karnataka, India	18, male, Sikhism	Undergraduate, no	Yes, offline
Adya (21:35 min)	Mumbai, Maharashtra, India	19, female, Hinduism	Undergraduate, no	Yes, online
Rohan (36 min)	Bangalore, Karnataka, India	20, male, Catholic Christianity	Undergraduate, no	Yes, offline
Babik (21:21 min)	Bangalore, Karnataka, India	20, male, Islam	Undergraduate, no	Yes, offline
Vahin (24:22 min)	Bangalore, Karnataka, India	22, male, Hinduism	Undergraduate, no	Yes, offline
Aditi (24:36 min)	Mohali, Punjab, India	18, female, Hinduism	Undergraduate, no	Yes, online

## Abbreviations

ERL: Emergency remote learning
IPA: Interpretative phenomenological approach
LMS: Learning management systems
SRL: Self regulated learning
